# Real-Time Tracking Data and Machine Learning Approaches for Mapping Pedestrian Walking Behavior: A Case Study at the University of Moratuwa

**DOI:** 10.3390/s24123822

**Published:** 2024-06-13

**Authors:** Harini Sawandi, Amila Jayasinghe, Guenther Retscher

**Affiliations:** 1 Department of Town & Country Planning, University of Moratuwa, Moratuwa 10400, Sri Lanka; harinisawandi@gmail.com (H.S.); amilabj@uom.lk (A.J.); 2 Department of Geodesy and Geoinformation, TU Wien—Vienna University of Technology, 1040 Vienna, Austria

**Keywords:** walking behavior, mobile GPS tracking, machine learning, pedestrian-friendly environment

## Abstract

The growing urban population and traffic congestion underline the importance of building pedestrian-friendly environments to encourage walking as a preferred mode of transportation. However, a major challenge remains, which is the absence of such pedestrian-friendly walking environments. Identifying locations and routes with high pedestrian concentration is critical for improving pedestrian-friendly walking environments. This paper presents a quantitative method to map pedestrian walking behavior by utilizing real-time data from mobile phone sensors, focusing on the University of Moratuwa, Sri Lanka, as a case study. This holistic method integrates new urban data, such as location-based service (LBS) positioning data, and data clustering with unsupervised machine learning techniques. This study focused on the following three criteria for quantifying walking behavior: walking speed, walking time, and walking direction inside the experimental research context. A novel signal processing method has been used to evaluate speed signals, resulting in the identification of 622 speed clusters using K-means clustering techniques during specific morning and evening hours. This project uses mobile GPS signals and machine learning algorithms to track and classify pedestrian walking activity in crucial sites and routes, potentially improving urban walking through mapping.

## 1. Introduction

Investigating pedestrian behavior and improving walking space in streets are becoming increasingly crucial considering the proven benefits to health, sustainability, and the development of safer pedestrian-friendly areas [1,2]. Consequently, an increasing amount of research has been carried out examining the relationship between the urban environment and individuals’ behavior on streets [3]. However, many of these studies focus on the macro level; in addition to considering the urban characteristics on a wider scale, it is important to also consider microscale factors of urban design that influence behavior on streets [1]. This requires collecting data on the micro-level walking behavior of individuals on the streets to obtain precise information and develop target solutions for promoting walking.

Behavior mapping is a commonly utilized technique for the direct and systematic monitoring of individual behaviors and locations [1]. This mapping was first used in indoor locations, primarily in the fields of psychology, sociology, and criminology. It has since become commonly employed in public spaces like streets, parks, and playgrounds [4,5]. Currently, this mapping extends to street design and street planning. Shoval et al. [6] utilized psychological mapping to generate real-time maps of subjective and objective emotions to analyze Jerusalem’s urban surroundings for the first time. Building upon that study, there is currently a growing interest in mapping behavior across several fields [7,8,9]. 

Recent technological advances have enabled these studies to use real-time surveying, tracking technologies, and global positioning systems (GPS). GPS data have been gathered since the 1990s to analyze transportation and access system performance, including measuring traffic flow, studying travel patterns, calculating route choices, etc. [10]. This opens a new research area by combining modern technical methods with real-time interactive communication. The availability of technology for the establishment of the geographic coordinates of mobile phones and other devices has significantly increased, leading to the emergence of a wide range of applications of location-based services (LBS) [11]. Similarly, Wi-Fi signals were also utilized in these studies [9]. The incorporation of LBS improves the quantitative metrics, offering significant spatial observation of the pattern of pedestrian movement. Smartphones, with their wireless connection, inertial sensors, and cameras, have greatly impacted digital health and gait analysis studies. Smartphones are currently providing physiological assessment and data entry/collection capabilities through several sensing modalities, which are accessible via apps [12].

However, this study found a few significant research gaps. The emergence of walking behavior depends on a combination of sociocultural variables, individual choices, and habitual patterns rather than a physical setting [13]. Therefore, to enhance our understanding of walking behavior patterns, we must acknowledge the complexity of this field.

Obtaining a detailed comprehension of individual experience in terms of time (second) and space (meters) offers new possibilities for study and strategic decision-making [6]. It is crucial to integrate the environment and individual characteristics of multiple individual inputs on the map to form a clear visual representation of different patterns [7]. The use of standard GPS positions is collected at intervals of a few seconds, resulting in hundreds of data points at each interval and large datasets for a thorough analysis. Thus, the first need is to map patterns of pedestrian walking behavior, as existing research highlights the importance of modern data gathering and analysis approaches, including objective walking patterns from large-scale tracking and machine learning analysis, to study the types of pedestrian walking patterns [14].

Most walking behavior studies have measured pedestrian walking utilizing walking duration, flows, and number of walkers [15]. Nevertheless, these studies are limited by their dependence on observational methodologies. Thus, while they offer new insights, their method has limitations [16]. Multiple authors identify the following five essential components in the observation process: 1. a visual representation of the observed areas; 2. a precise explanation of the human behavior observed, tracked, described, or outlined; 3. a timetable of recurring intervals for observation and recording; 4. a methodological observation process; and 5. a system for programming analysis that reduces the recording workload [1]. While walkability studies highlight the complexity of walking activities, there is still a lack of systematic categorization of pedestrian actions, creating a knowledge gap [9]. Further, research on walking patterns disregards the individual travel direction and time of the day. The individual travel direction has a significant impact on walking behavior changes; in addition, the direction in which people walk is influenced by built environment scenarios [17]. Also, time series analysis can be used to study changes in behavior over time [8]. The absence of this information presents difficulty in acquiring walking patterns. Hence, the second need involves considering individual travel direction and temporal factors to map and analyze walking behavior using real-time tracking applications. This approach allows researchers to collect more detailed and accurate data, resulting in more effective urban planning efforts.

The behavior of human walking is naturally complex and shaped by a variety of environmental and psychological factors. Subjective verification, which is frequently based on personal user experience and perception, plays a crucial role in validating the accuracy of measurement models, ensuring that they accurately reflect the complex nature of real-world walking scenarios. Current research on evaluating human walking behavior primarily concentrates on the construction of index systems, as well as data collecting and processing. However, it does not include any verification of the reliability of measurement findings or the validity of measurement models [18].

In this context, analyzing pedestrian walking patterns using machine learning algorithms is critical for improving road safety and optimizing traffic management. Traditional techniques, such as artificial neural networks (ANNs) and hidden Markov models (HMMs), have been widely used in this domain [19]. However, these algorithms frequently encounter significant issues, such as overfitting and substantial data dependency, in real-time processing scenarios. For example, Ajmaya and Eklund’s [19] study on recognizing pedestrian events using IMU and GPS data emphasized the challenges of improving ANN models, as well as the vast amount of training data required to obtain reliable results. Furthermore, the study by Gong et al. [20] identified limitations in using density-based spatial clustering of applications with noise (DBSCAN-TE) and support vector machine (SVM) methodologies, such as the need for parameter estimation and the lack of instant speed and acceleration features, which reduce the accuracy and efficiency of detecting pedestrian stops and movements. 

Although data-driven methods have the potential to assist in making informed design decisions, it is still uncertain which new sources of information and approaches could be utilized to obtain insights into studying pedestrian walking behaviors in urban areas, resulting in a shortage of knowledge. Given the focus of our work, machine learning approaches have also been utilized in various areas of GNSS positioning. For instance, Zhang et al. [21] used unsupervised machine learning techniques to enhance precise positioning and navigation in complex environments. By integrating best integer equivariant (BIE) estimation with unsupervised K-means clustering algorithms, the proposed methods significantly improved both accuracy and reliability. The experiment demonstrates that the use of this approach could achieve millimeter-level precision, highlighting the effectiveness of machine learning techniques in such applications. In this study [21], K-means achieved high accuracy rates for inferring transportation modes, particularly when speed profiles were used as attributes. This implies that K-means clustering can handle the spatial and temporal patterns in pedestrian movement data without considerable parameter modification. 

Given the limitations and potentialities in current studies, this research developed a framework utilizing unsupervised machine learning techniques to map pedestrian walking behavior in streets with real-time tracking data using mobile phones. This research endeavors to explore pedestrian walking behavior by utilizing mapping techniques to provide insight that exceeds traditional methods. Researchers can collect complex characteristics of pedestrian behavior, such as movement patterns, and interaction with the built environment using real-time tracking technologies and advanced mapping approaches. Integrating tracking technologies with subjective and objective experiences of human behavior could greatly enhance urban planning [6]. Extensive mapping not only helps to improve urban design to promote pedestrian activity but also creates convenient urban environments.

## 2. Materials and Methods

### 2.1. Case Study 

**Figure 1:**
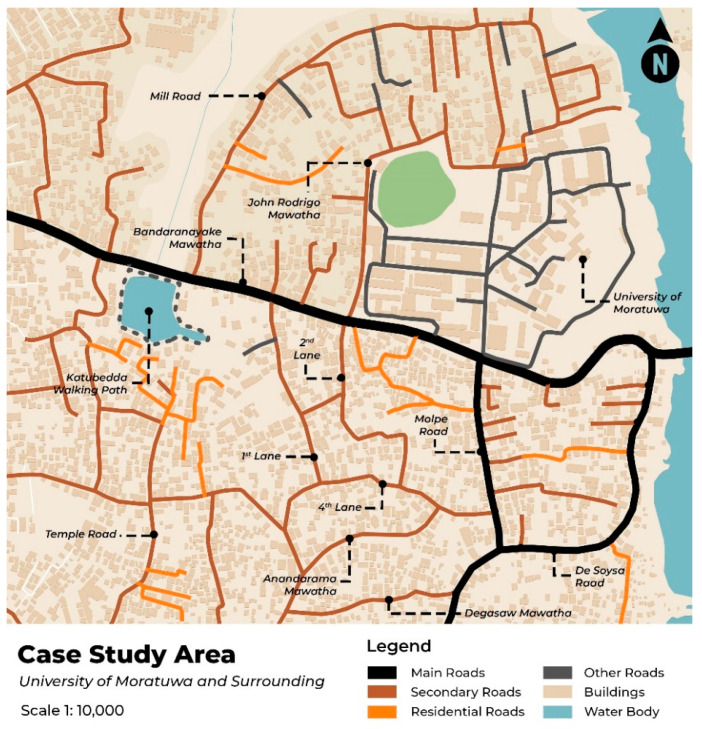
Case study area.

We developed an approach for mapping pedestrian walking behavior on the streets of the University of Moratuwa and its vicinity. Campus walking areas are prioritized in urban sustainable development and developing pedestrian-friendly surroundings [2]. Given the nature of the experiment, this study examined walking behavior at the University of Moratuwa and its surrounds, as shown in Figure 1. The case study includes public locations, residential neighborhoods, cultural and commercial areas, and diverse university spaces to evaluate walking behavior patterns in varied environments and situations. Pedestrian traffic was seen in these areas, as many individuals were walking around the university because university students choose different routes daily to get to the university.

### 2.2. Experimental Design and Workflow 

University students who live near the University of Moratuwa were selected as a sample. These students, aged 23 to 27 years, were chosen for this study because they were well-acquainted with their surroundings due to their daily commuting from their accommodation (boarding) to the university. Initially, 60 individual samples with a 50% gender distribution were chosen. The experiment was only carried out on weekdays. Weekday tests allowed for more focused monitoring of university pedestrian behavior. Specific times for the experiments were set to prevent overcrowding and congestion. The times were 7:00 to 9:00 a.m. and 4:00 to 6:00 p.m. These times of the day were carefully chosen to include both morning and evening peak hours to thoroughly examine pedestrian behavior. 

**Figure 2:**
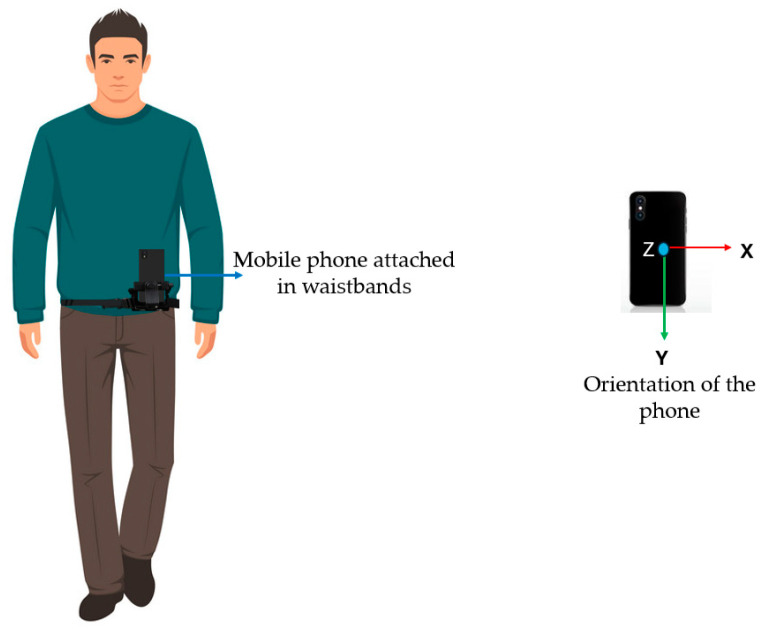
Experiment setup.

As pointed out in the study by [18,22], which investigated several accelerometer placements, including at the hip (belt), wrist, upper arm, ankle, and thigh of the test person, using numerous accelerometers aids in activity identification. Thus, as illustrated in Figure 2, in this study, participants were advised to secure their mobile phones to their waistbands with designated holders. This strategy guaranteed that the phones remained in a consistent and steady position throughout the data collection process. The test persons were advised to keep the mobile phone in a vertical orientation within the holder to preserve consistency. This placement of the phones enabled the accurate gathering of data on walking speed, acceleration, and GPS position during the experiments. 

Also, to effectively capture speed data when walking, the following parameters needed to be considered: holding the phone upright in portrait orientation by ensuring that the X-axis (horizontal) is parallel to the direction of movement while the Y-axis (vertical) is perpendicular to the ground. This orientation enables the phone’s sensors to better capture forward movement (along the X-axis) and up-and-down motion (along the Y-axis), both of which are important for estimating speed.

Data for this study were collected using the Redmi Note 12 Pro smartphone, which has a powerful sensor suite that includes an accelerometer, gyroscope, and GPS receiver. Throughout the experiment, the use of these sensors was of utmost importance for collecting data on walking speed, acceleration, and geographic positions. Furthermore, the smartphone’s long-lasting battery allowed for continuous data collection over lengthy periods.

Each participant was instructed to use their mobile phone to record their walking speed, acceleration, and other variables available through the mobile app. Over four weeks, participants were required to record these characteristics as they traveled around the case study area during both the morning and evening. To minimize bias from long-distance walking and associated fatigue, each recording session was limited to 10 min. Accurate instructions on how to carry the phones were provided to participants. To ensure precise data collection, participants were additionally directed not to check their phones while walking and to keep focused on their surroundings. Every participant was informed of this study’s purpose and the possible results, and they were all given specific instructions to explore and experience the environment throughout the study walking sessions. Throughout the process of this research, ethical standards and privacy have been carefully upheld.

### 2.3. Data Analysis 

Clustering individuals based on their walking speed, direction, and time enables the precise classification of walking behavior. K-means clustering aids in identifying clusters [9]. Mapping identifies locations with high levels of pedestrian traffic and their distribution. Clustering identifies areas with high levels of foot traffic or congestion hotspots, allowing for more analysis to guide more effective solutions. Data analysis included three steps.

#### Framework of This Study

**Figure 3:**
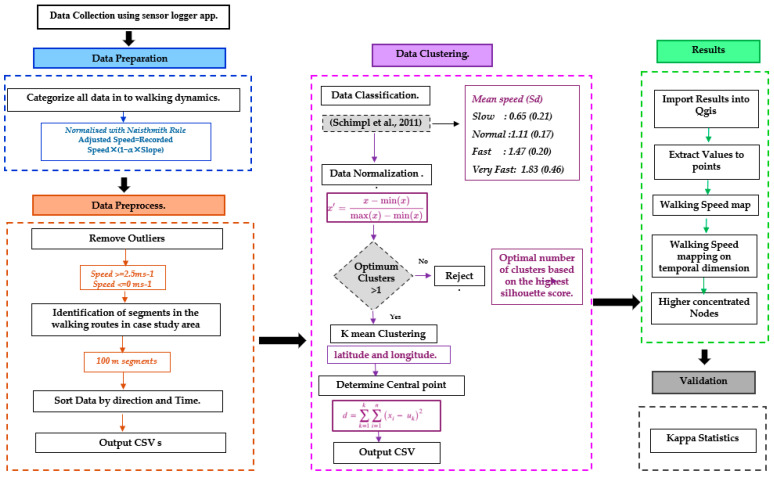
Framework of this study. Framework of this study. The data classification component under the data clustering is based on the methodology proposed by Schimpl et al. [23].

Figure 3 illustrates the framework used for evaluating location data and analyzing pedestrian walking behavior. This framework offers a structured approach to gaining insights from the dataset.

- Data collection using the sensor logger app;
- Data preparation—Before beginning the data preprocessing, each CSV file is classified based on the time of travel and travel direction to gain additional insights for a comprehensive dataset;
- Data preprocessing—The initial phase of the farmwork involves manual data preprocessing;
- Data preparation for K-means clustering—The dataset was cleaned and normalized to detect clusters of pedestrian walking behavior. We used a bespoke algorithm to preprocess the dataset;
- Mapping the results—The work principally centers on cluster analysis, employing unsupervised machine learning methods to reveal noteworthy trends and identify the homogeneous profile among pedestrians;
- Data validation—Data validation is conducted through the outputs of mapping using K-means clustering and expert subjective assessment.

1. Data collection using sensor logger app.

**Figure 4:**
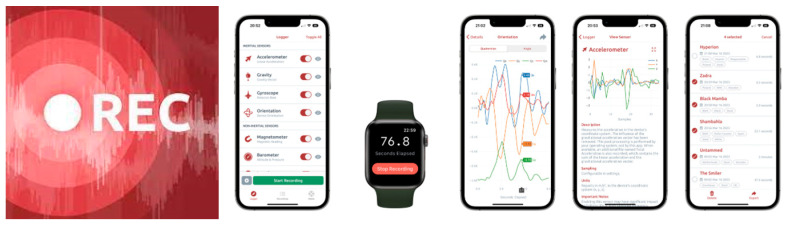
Sensor logger mobile application.

Sensor logger is a smartphone app that collects objective participant data. Figure 4 illustrates the interface of the sensor logger mobile application, which is used to gather data. The application is available for Android, iPhone, and Apple Watch. The main reason for choosing this mobile app is its ability to adjust walking speed according to the Naisthmith rule, which sets it apart from other apps. It was useful for tracking small speeds caused by terrain changes when walking. Also, by using this mobile app, one can capture diverse walking dynamics. It mostly records the participant’s geographical location and timestamp as they walk along the path. The program records acceleration, location, gyroscope, speed, step count, sound, heart rate, wrist motion, and other elements to capture the walking behavior. While the sensor logger app collects a range of data types, this analysis prioritizes the use of metrics appropriate to this study’s aims. We specifically focus on using location data in conjunction with metrics like walking speed, longitude, latitude, accuracy, time, etc. In this mobile application, speed is calculated by measuring the change in consecutive GPS coordinates over time. The application determines speed by recording latitude and longitude at each time point and calculating the distance walked throughout each interval. The app’s privacy practices may involve the management of subsequent data. The data can be exported in several forms, such as Zip, CSV, JSON, and SQLite.

The entire dataset was initially normalized using the Naisthmith method to ensure consistency in the measurements of walking speed across various terrains. The Naisthmith rule is a common technique for calculating the actual working speed when facing slopes or uneven surfaces.

The Naismith rule can be described using the following:

Equivalent distance = Horizontal distance + (Vertical distance ∗ α)(1)

The horizontal distance refers to the distance at which an object moves on a level surface, whereas the vertical distance indicates the rate of ascent or descent. The parameter α represents Naisthmith’s number, a constant coefficient that quantifies the additional exertion needed as a result of variations in altitude, which is usually set to 7.92.

2. Data Preparation

The data collected includes multiple characteristics, such as time, elapsed seconds, longitude, latitude, altitude, speed, bearing accuracy, vertical accuracy, horizontal accuracy, and bearing. The dataset chosen for this study is presented in Table 1. The mobile data covers 4 weeks and includes a complete dataset of 96,924 points. Before starting the data preprocessing, each CSV file based on time travel and travel direction has to be classified to gain additional insights for a comprehensive dataset.

3. Data preprocessing

The dataset included individual speed points captured using speed-tracking techniques in the mobile phone app. Following the framework proposed, an initial phase in the data preprocessing was the removal of outliers from each user’s output. Outliers are samples that appear to be inconsistent with the overall trend of the GPS signal. They could be peaks, discontinuities, saturation, etc. To properly assess a signal, it must be removed without affecting the rest of the data. In the context of analyzing walking behavior patterns, outliers were defined as speed values outside the normal walking range, as follows: a speed of 0.0 ms^−1^, indicating negligible movement, such as waiting, and a speed of 2.5 ms^−1^ or more, which is likely inaccurate due to GPS signal errors. 

In addition to outlier removal, the dataset was analyzed for direction of travel and time. The manual process involved analyzing each user’s outputs for GPS points and assigning the relevant direction based on timestamps using QGIS. 

When assessing GPS signals for walking behavior, it is critical to focus on potential outliers induced by rapid and major shifts in the signal, such as significant braking. Before running the algorithm, the outlier removal strategy was used to thoroughly evaluate the data for outliers and ensure the correct classification of valid samples.

4. Data preparation for K-means clustering

Clustering techniques are divided into types based on splitting, density, and model. The K-means algorithm offers several advantages over other established approaches, such as straightforward mathematical principles, quick convergence, the improved scalability to big datasets, the effective management of high-dimensional datasets, and straightforward implementation. This approach is adaptable and can be utilized across several fields, as well as simply adapted to new scenarios. The reason for choosing K-means clustering is its capability to cluster data by reducing the sum of squared error (SSE) inside clusters.The sum of squared error (SSE) is given by the following equation:

(2) $$d=\sum_{k=1}^{k}\sum_{i=1}^{n}{\left(x_{i}-u_{k}\right)}^{2}$$

The main function of the sum of the squared error is represented by d, where k is the number of clusters, n is the number of observations, $x_{i}$ is an observation i, and $u_{k}$ is the centroid generated for the cluster of $x_{i}$. 

However, typical K-means clustering has limitations. To address these issues in this unsupervised machine learning model, a framework has been developed by following specific techniques.

- Utilizes only numerical input variables—K-means uses distance-based metrics to analyze the similarity between data points, restricting the evaluation to only numerical factors. The analysis utilized geographic longitude and latitude coordinates to identify the pattern of walking behavior. In addition, the undefined (NaN) values were removed. Clustering results may be distorted if NaN values are included in the raw dataset (CSV output);
- Outlier removal in data classification—To cluster the data for studying walking behavior, the major dynamic being considered here is walking speed, which was collected through a mobile application. To categorize the speed of data, the existing literature has been examined. The speed property divides walking speeds into the following four categories: “Slow”, “Normal”, “Fast”, and “Very Fast” [23]. It is imperative to evaluate the potential impact of outlier data on the K-means clustering analysis during the preparatory phase at this stage. The IQR-based outlier removal method was used on each speed category to remove data points that were outside the permitted range. Table 2 shows the average value of accuracy in each cluster after removing the outliers and categorizing them into clusters.

- Data Normalization—Data normalization was performed using the min–max scaler method [24] in Python using the sci-kit-learn package. The min–max scaler is given as follows:
  (3) $$x^{1}=\frac{x-\min\left(x\right)}{\max\left(x\right)-\min(x)}$$
  Let $x^{1}$ represent the normalized value, x represents the initial value within a particular range, min⁡(x) represents the minimum value of the attribute within that range, and max⁡(x) represents the maximum attribute value within that range.
  This phase ensured that the results were not affected by variations in scales and that all scales had an equal impact on model fitting.
- The optimal number of clusters—This study utilized the K-means method to identify unique patterns in the data based on geographical coordinates (latitude and longitude) and walking speeds. Clustering algorithms depend on a random initialization of the cluster centroid. Silhouette analysis (SA) was used to address the issue and determine the ideal number of clusters for each speed category [25]. Introduced by Rousseeuw in 1987, the silhouette analysis (SA) technique calculates the silhouette score, a statistic that varies between −1 and 1. This score provides information on the proximity and density of clusters, indicating their closeness or distance from one other and the total density of the clusters.

5. Mapping the Results

The silhouette analysis (SA) approach helped to identify the ideal number of clusters within each speed category. The next step was to map these clusters to reveal distinctive patterns within the case study area. Basic QGIS mapping techniques were used to accomplish this.

6. Data Validation

Validating the results is essential after finishing the analytical process. Validating these results is challenging because spatial quality is a normative criterion [26]. This study used a unique method that contrasted the results of the unsupervised machine learning algorithm with the preferred speed of each cluster as identified by the students participating in the research.

For this method, 150 clusters, which is one-third of all cluster setups, were carefully chosen for assessment. These clusters were specifically used because they were within one standard deviation. A total of 150 pictures were manually captured during the evaluation procedure in the study area. Travel direction and traveled time were considered for each cluster when taking the photos. The research participants were required to walk in a specific direction and only identify what was directly in front of them; thus, the analysis disregards the whole 360-degree field of vision and instead focuses on a 120-degree field of view based on their walking path. 

The camera features an 8-megapixel resolution and an f/2.2 aperture, ensuring satisfactory image detail and effective light capture, respectively. Featuring a 120-degree ultrawide field of vision, this device is capable of effectively capturing wide and expansive scenes as well as group photographs. Furthermore, the camera’s 1/4.0-inch sensor size and 1.12 µm pixel size is advantageous. Students were asked to rate their preferred speed of walking when looking at images categorized as slow, normal, fast, and very quick. 

## 3. Results

### 3.1. Results of Cluster Mapping 

By using the K-means clustering techniques, a total of 622 speed clusters were identified during both morning hours (07.00 to 09.00 a.m.) and evening hours (4.00 to 06.00 p.m.) independently. The results discussed below are based on collected data and represent the areas most extensively used by students. GIS and spatial analysis are employed to map the results of the clustering of walking behavior, which includes point distribution maps that illustrate pedestrian concentration on the road.

**Figure 5:**
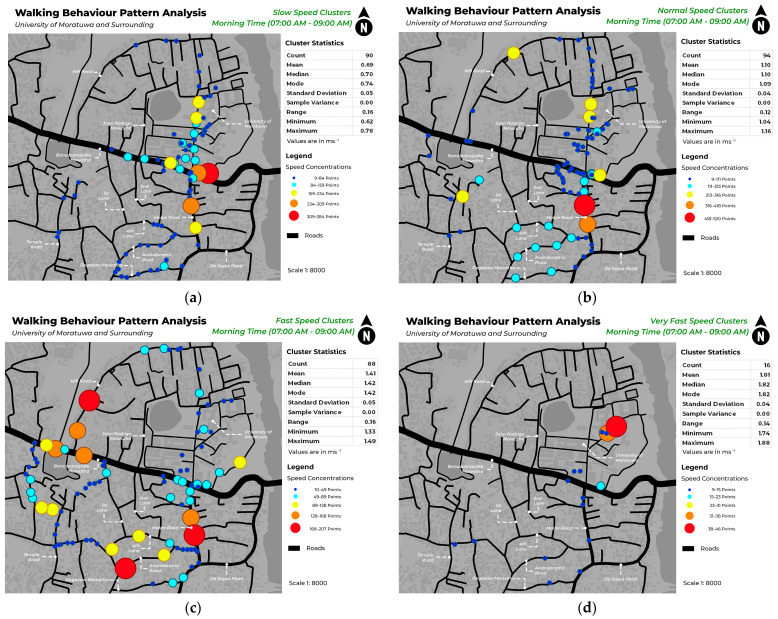
Pedestrian concentration in slow walking speeds (**a**), normal walking speeds (**b**), fast walking speeds (**c**), and very fast walking speeds (**d**) in the morning hours.

**Figure 6:**
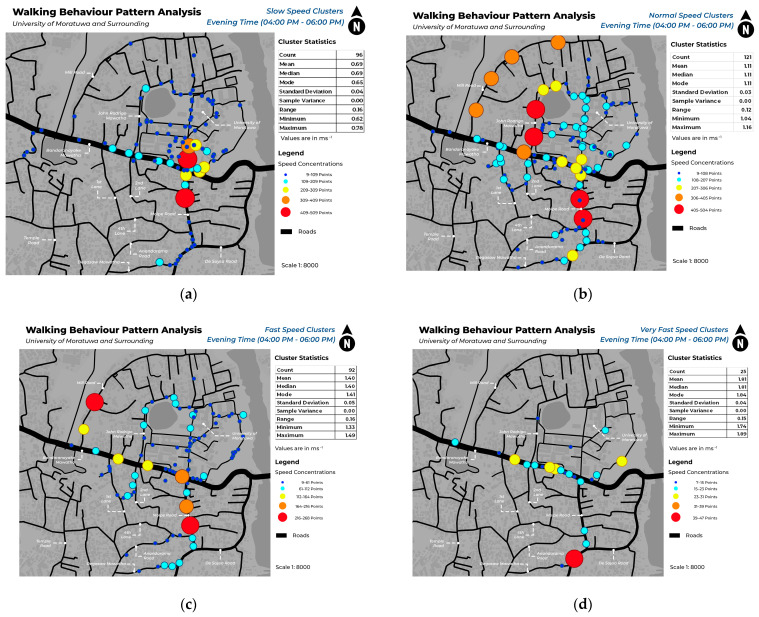
Pedestrian concentration in slow walking speeds (**a**), normal walking speeds (**b**), fast walking speeds (**c**), and very fast walking speeds (**d**) in the evening hours.

**Figure 7:**
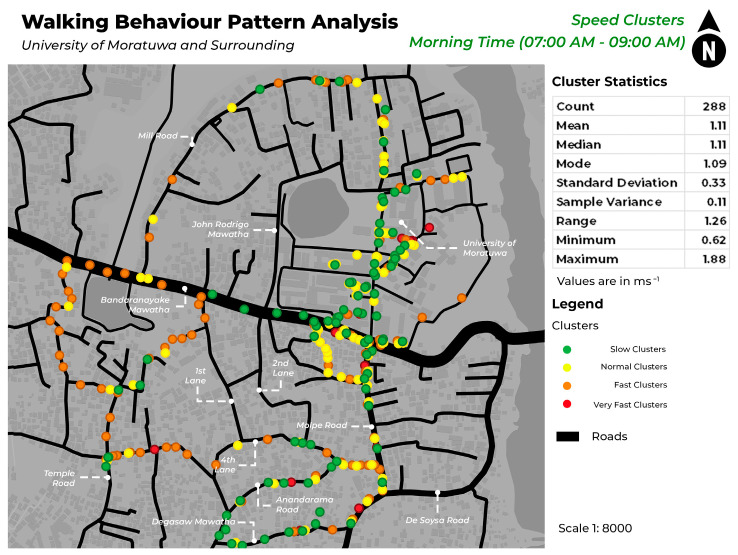
Pedestrian concentration in different walking speed ranges in the morning hours.

**Figure 8:**
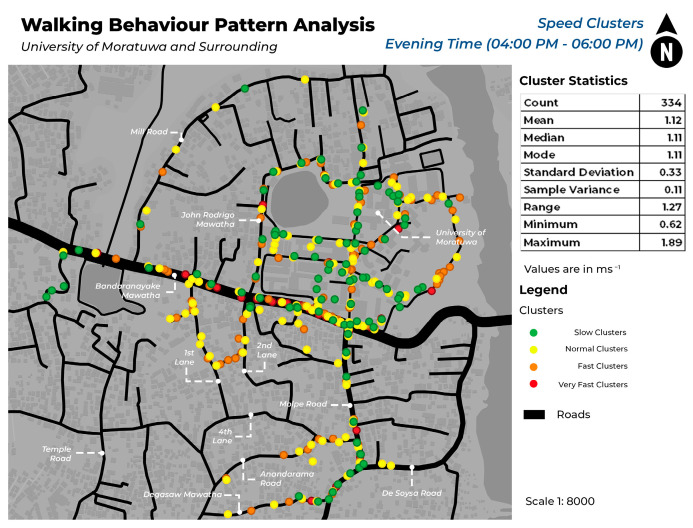
Pedestrian concentration in different walking speed ranges in the evening hours.

This study mapped pedestrian density in various locations within each of the four speed categories during morning and evening hours. Figure 5 illustrates the spatial distribution of pedestrian density among different speed clusters in the morning, whereas Figure 6 illustrates pedestrian concentration in the evening. The identification of unique spatial behaviors within each cluster is facilitated by the display of data in both morning and evening timestamps, which enables the observation of a variety of patterns. Figure 7 and Figure 8 depict the walking behavior patterns in the morning and evening based on cluster analysis. The points indicate the centroid of each cluster derived from K-means clustering. 

### 3.2. Results of Data Validation 

Appendix A shows the results of ratings obtained by machine learning and the recordings from the students. For each of the 150 clusters, two ratings were estimated. Out of the 150 ratings, 126 were found to be the same, which is considered acceptable. The results were assessed for validity using Kappa Statistics to quantify the inter-rater reliability between the ratings made using machine learning and the recordings from the students. The values were calculated using Python. Table 3 presents the summary of the results. 

## 4. Discussion

This research enhances the understanding of the complex connections between urban environments and pedestrian behavior by utilizing quantitative location data and speed data of pedestrians, along with qualitative validation of the findings. “Big Data” analysis is used to process and analyze significant and complicated datasets that typical data processing systems are not able to handle. Various methods exist for gathering and analyzing vast amounts of data, but there is a need for a standardized framework to extract insights. In this paper, a method is proposed for conducting experiments using the pedestrian to collect and analyze data and extract insights to conclude behavior, rather than relying on biased observational data or the existing literature.

This study’s findings provide useful insights into pedestrian behavior patterns within various speed categories. The clustering study using unsupervised machine learning discovered several clusters for each of the following speed categories: slow, normal, fast, and very fast. The clusters indicate regions with different pedestrian densities and speeds of movement. Mapping highlights locations with a significant concentration of each cluster on different roadways. The slow mean walking speed is 0.69 ms^−1^, the normal mean walking speed is 1.11 ms^−1^, the fast mean speed is 1.41 ms^−1^, and the very fast mean speed is 1.81 ms^−1^.

In this case, both in the morning and evening hours, a significant increase in pedestrian traffic on Bandaranayake Mawatha and Molpe Road can be observed. In those areas, there is a high concentration of slow and normal walking pedestrians. The field observations show that these locations correspond to the main entry for vehicles at the University of Moratuwa, where there is a notable rise in vehicular traffic and dense retail structures along Molpe Road. 

According to a study by [27], young adults walk at a speed of 89 m/min in educational areas and 80 m/min in commercial areas. According to the speed ranges in this study, the speed falls within the normal range. Results show that pedestrians passing around the university area (Bandaranayake Mawatha) and the nearby commercial area (Molpe Road) tend to walk at a normal speed, which aligns with the existing findings of the aforementioned literature. 

Inside the University of Moratuwa, during the morning hours, there is a high degree of pedestrian concentration observed in the Lagan area. This congestion can be categorized as slow and normal speed clusters. Based on on-site observation, this location in the university is predominantly a green environment, which is similar to findings of [28] research that indicates that individuals tend to walk slower in greener environments. In addition, the concentration of speed in different areas of the University of Moratuwa is constant. 

This study found a substantial correlation (85%) between machine-generated scores and subjective assessments. This verification demonstrates the effectiveness of our strategy, which benefits substantially from the large dataset employed for analysis.

Finally, using the experimental study, we were able to visualize, accurately analyze, and validate walking behavior patterns. Visualization is essential for managing tracking data. GPS is important in urban research because it provides precise and observable data that can be combined to create a new evidence base for predicting future urban trends.

## 5. Conclusions and Outlook

The objective of this research is to present an approach for analyzing pedestrian paths in urban environments. Individuals’ walking patterns are evaluated on multiple streets inside the case study location. Our employed method provides a full understanding of pedestrian dynamics by gathering real-time tracking data and using unsupervised machine learning methods to assess walking behavior. 

This work provides insights into walking behaviors. Firstly, this study adds to the current body of knowledge by developing a theoretical basis for using unsupervised K-means clustering machine learning algorithms to assess pedestrian walking behavior using large-scale GPS data. Secondly, our work expands the transportation planning and urban design and planning literature by using large-scale data analysis with established methodologies. 

The authors’ analysis of outcomes utilizing machine learning algorithms and mobile location data collection led to the following conclusions and interpretations. K-means clustering was used to determine the number of unique clusters within the case study area. This method was tested at various times of day and discovered that there is a different pattern in morning and evening walking behavior, demonstrating constancy in pedestrian concentration in the studied area. These patterns may arise as a result of urban design components and activities in an area that influences pedestrian movement patterns. Therefore, it is recommended in future research to analyze the spatial and environmental aspects of the study region to enrich the existing body of knowledge.

This study, however, is limited by continuous data collection due to technical challenges, resulting in missing information on certain days. This study only collected pedestrian movement data for a limited number of days and had a small sample size comprising only university students. The primary objective of this work was to examine the suggested methodology and showcase its efficacy in real-time tracking data by employing machine learning techniques to analyze pedestrian locomotion patterns. This study was conducted across two time periods, focusing on temporal aspects. Future research could benefit from extending the duration of observations, analyzing the temporal patterns of different activities in depth, and studying the diverse street users. 

## Figures and Tables

**Table 1:** Types of data collected for this study.

Second Elapsed (Seconds)	Bearing Accuracy (Degrees)	Speed Accuracy (ms^−1^)	Vertical Accuracy (m)	Horizontal Accuracy (m)	Speed (ms^−1^)	Bearing (Degree)	Altitude (m)	Longitude (Degrees)	Latitude (Degrees)
4188.1	0	0.0806	34.74	9.94	0.0001	0	−77.2	79.9002	6.7956
2096.2	0	0	4.18	11.70	0.0001	0	−76.4	79.8990	6.7953
1699.1	0	0.15	1.13	13.32	0.0002	0	−73.8	79.9000	6.7963

**Table 2:** Average values of each cluster used in K-means clustering.

Cluster	Bearing Accuracy (Degrees)	Speed Accuracy (ms^−1^)	Vertical Accuracy (m)	Horizontal Accuracy (m)	Speed (ms^−1^)
Slow	0	0	0.259	0.543	0.6775
Normal	0	0.1485	0	0.600	1.1081
Fast	0	0	0	0.677	1.4091
Very Fast	0	0	0.548	0.667	1.5447

**Table 3:** Statistics of the machine learning and the recordings from the students indicating the agreement value.

Agreement	Cohen’s Kappa Coefficient	Std. Err.
85.3%	0.8	0.013

## Data Availability

The data supporting the reported results were generated during the data collection phase of this study. Due to privacy and ethical restrictions, the data are not publicly available.

## Appendix A

**Table A1:** Summary table of the evaluation using machine learning and subjective evaluation conducted by the students.

Cluster No	Machine Evaluation	Subjective Evaluation	Cluster No	Machine Evaluation	Subjective Evaluation
1	Very Fast	Very Fast	76	Fast	Fast
2	Very Fast	Very Fast	77	Normal	Normal
3	Very Fast	Fast	78	Fast	Fast
4	Very Fast	Fast	79	Fast	Fast
5	Fast	Fast	80	Fast	Fast
6	Very Fast	Very Fast	81	Normal	Fast
7	Very Fast	Very Fast	82	Very Fast	Fast
8	Normal	Normal	83	Fast	Normal
9	Very Fast	Fast	84	Slow	Slow
10	Very Fast	Very Fast	85	Normal	Normal
11	Very Fast	Very Fast	86	Fast	Fast
12	Slow	Normal	87	Very Fast	Very Fast
13	Slow	Slow	88	Very Fast	Fast
14	Fast	Fast	89	Fast	Fast
15	Slow	Slow	90	Very Fast	Very Fast
16	Very Fast	Very Fast	91	Fast	Fast
17	Normal	Normal	92	Slow	Slow
18	Normal	Normal	93	Normal	Fast
19	Fast	Fast	94	Fast	Fast
20	Fast	Very Fast	95	Very Fast	Very Fast
21	Very Fast	Very Fast	96	Slow	Slow
22	Fast	Fast	97	Fast	Fast
23	Normal	Normal	98	Normal	Normal
24	Fast	Fast	99	Slow	Slow
25	Slow	Slow	100	Slow	Slow
26	Fast	Fast	101	Normal	Fast
27	Slow	Slow	102	Normal	Normal
28	Slow	Slow	103	Normal	Normal
29	Fast	Fast	104	Fast	Fast
30	Normal	Normal	105	Slow	Slow
31	Normal	Normal	106	Normal	Normal
32	Normal	Normal	107	Normal	Normal
33	Normal	Normal	108	Slow	Slow
34	Normal	Normal	109	Very Fast	Very Fast
35	Normal	Normal	110	Slow	Slow
36	Very Fast	Very Fast	111	Very Fast	Very Fast
37	Very Fast	Fast	112	Fast	Fast
38	Fast	Fast	113	Fast	Fast
39	Fast	Normal	114	Fast	Fast
40	Slow	Slow	115	Fast	Fast
41	Slow	Slow	116	Slow	Slow
42	Very Fast	Very Fast	117	Slow	Normal
43	Slow	Slow	118	Fast	Fast
44	Very Fast	Very Fast	119	Slow	Slow
45	Slow	Slow	120	Normal	Slow
46	Normal	Normal	121	Normal	Normal
47	Slow	Slow	122	Normal	Normal
48	Fast	Fast	123	Slow	Slow
49	Normal	Normal	124	Slow	Slow
50	Slow	Normal	125	Slow	Slow
51	Normal	Normal	126	Normal	Normal
52	Slow	Slow	127	Slow	Normal
53	Very Fast	Very Fast	128	Normal	Normal
54	Normal	Normal	129	Normal	Normal
55	Slow	Slow	130	Very Fast	Normal
56	Very Fast	Very Fast	131	Slow	Fast
57	Slow	Slow	132	Very Fast	Very Fast
58	Normal	Normal	133	Very Fast	Very Fast
59	Normal	Normal	134	Slow	Slow
60	Fast	Fast	135	Slow	Slow
61	Slow	Slow	136	Normal	Normal
62	Normal	Normal	137	Fast	Very Fast
63	Normal	Slow	138	Normal	Normal
64	Slow	Slow	139	Fast	Very Fast
65	Slow	Slow	140	Slow	Slow
66	Slow	Slow	141	Very Fast	Very Fast
67	Very Fast	Very Fast	142	Slow	Slow
68	Normal	Normal	143	Very Fast	Very Fast
69	Very Fast	Very Fast	144	Very Fast	Very Fast
70	Normal	Normal	145	Normal	Normal
71	Normal	Fast	146	Normal	Normal
72	Slow	Slow	147	Normal	Normal
73	Very Fast	Very Fast	148	Normal	Normal
74	Fast	Fast	149	Fast	Normal
75	Very Fast	Very Fast	150	Fast	Fast
